# Nerve Root Compression Analysis to Find Lumbar Spine Stenosis on MRI Using CNN

**DOI:** 10.3390/diagnostics13182975

**Published:** 2023-09-18

**Authors:** Turrnum Shahzadi, Muhammad Usman Ali, Fiaz Majeed, Muhammad Usman Sana, Raquel Martínez Diaz, Md Abdus Samad, Imran Ashraf

**Affiliations:** 1 Department of Information Technology, University of Gujrat, Gujrat 50700, Pakistan; turrnumshahzadi@gmail.com (T.S.); fiaz.majeed@uog.edu.pk (F.M.); m.usman@uog.edu.pk (M.U.S.); 2 Department of Computer Science, University of Gujrat, Gujrat 50700, Pakistan; m.usmanali@uog.edu.pk; 3 Universidad Europea del Atlántico, Isabel Torres 21, 39011 Santander, Spain; raquel.martinez@uneatlantico.es; 4 Universidad Internacional Iberoamericana, Campeche 24560, Mexico; 5 Universidad Internacional do Cuanza, Cuito EN250, Bié, Angola; 6 Department of Information and Communication Engineering, Yeungnam University, Gyeongsan 38541, Republic of Korea

**Keywords:** lumbar spine stenosis, magnetic resonance imaging, deep learning, image processing

## Abstract

Lumbar spine stenosis (LSS) is caused by low back pain that exerts pressure on the nerves in the spine. Detecting LSS is a significantly important yet difficult task. It is detected by analyzing the area of the anteroposterior diameter of the patient’s lumbar spine. Currently, the versatility and accuracy of LSS segmentation algorithms are limited. The objective of this research is to use magnetic resonance imaging (MRI) to automatically categorize LSS. This study presents a convolutional neural network (CNN)-based method to detect LSS using MRI images. Radiological grading is performed on a publicly available dataset. Four regions of interest (ROIs) are determined to diagnose LSS with normal, mild, moderate, and severe gradings. The experiments are performed on 1545 axial-view MRI images. Furthermore, two datasets—multi-ROI and single-ROI—are created. For training and testing, an 80:20 ratio of randomly selected labeled datasets is used, with fivefold cross-validation. The results of the proposed model reveal a 97.01% accuracy for multi-ROI and 97.71% accuracy for single-ROI. The proposed computer-aided diagnosis approach can significantly improve diagnostic accuracy in everyday clinical workflows to assist medical experts in decision making. The proposed CNN-based MRI image segmentation approach shows its efficacy on a variety of datasets. Results are compared to existing state-of-the-art studies, indicating the superior performance of the proposed approach.

## 1. Introduction

Lumbar spine stenosis (LSS) is a severe back pain disease caused by a degenerative process that compresses the spinal cord and exiting nerve roots, also known as central and foraminal or lateral stenosis [1]. The lumbar contains five vertebrae labeled L1 to L5, the different areas of which may be affected by chronic low back pain (CLBP). CLBP can be caused by a number of factors, including fractures, lumbar disc degeneration, lumbar disc herniation, or infection of the nerve roots. Any of the factors listed above might lead to LSS; however, CLBP is a general term used to define the LSS cause [2]. CLBP negatively affects the health of millions of people around the globe, severely disturbing and employment, personal, and social lives [3]. According to statistics reported in [4], 50% to 80% of adults experience LBP at some point in their lives, representing the most prevalent illness in the world.

CLBP is a frequent consequence of a less serious but acute type of LBP. If the underlying cause is significant and ignored, LBP can develop from acute to chronic status [5]. Acute back pain lasts for a few days to weeks and can be resolved by self-care, as it can last for 12 weeks or longer [6]. According to a National Health Service (NHS) study of the economic impact of CLBP in the United Kingdom (UK) [7], the probability of a patient’s rehabilitation success is primarily based on a timely diagnosis of the LBP cause. Verbiest [8] proposed the term neurogenic claudication to characterize the symptoms that individuals with CLBP experience. Numbness, coldness, burning, and cramping are a few symptoms of the ailment. LBP might begin in the buttocks and extend to the thigh and leg. LSS is a narrowing of the spinal column or vertebral foramina that puts pressure on the thecal sac and posterior nerve roots, either directly or indirectly [9].

An LSS diagnosis is usually carried out utilizing imaging modalities including magnetic resonance imaging (MRI), radiographic myelography (RM) scans, and intravascular ultrasound (IVUS). X-rays can only show the bony part of the spine; soft tissue structures such as the intervertebral discs, muscles around the spine, spinal cord, and spinal nerves cannot be directly evaluated by X-rays alone. MRI produces multiple images of different views that can help provide clearer pictures for the doctor to analyze. Another approach for computer-aided medical diagnosis is the use of IVUS images, which has been well recognized as a powerful imaging technique to evaluate stenosis inside the coronary arteries. These approaches can not only relieve the burden on radiologists but also increase the certainty of a precise diagnosis. MRI is much more common [10] in hospitals than other imaging modalities such as RM and IVUS. MRI is more ubiquitous, since it is the only technique for back pain diagnosis [11], while RM detects the most challenging cases of LSS. A lumbar spine MRI of a patient can be viewed in two ways: from a sagittal (side) or axial (top-down) view [12].

A major source of concern is the fact that in the previous decade, there has been a significant shortage of neuroradiologists [13]. As a result, early disease detection may not always be achievable, since diagnosis may take several weeks due to the need to obtain a referral and the time it takes to see a medical specialist and perform medical scans and analysis. A report from the Royal College of Radiologists [14] showed that three-quarters of UK medical imaging departments lack radiology specialists to provide healthcare services, resulting in increased spending on outsourcing, overtime, and expert doctors to substitute radiologist duties each year. Since 1995, the demand for radiographic imaging, including MRI and computed tomography (CT) scans, has increased at an average annual rate of 12.3%. As a result, improved methods for computer-aided diagnosis (CAD) systems are required to extract diagnostic results from MRI and other modalities. Due to a lack of time to study a case, a radiologist may overlook a disease that a CAD system may detect. This insight drives us to improve the efficiency and effectiveness of automated lumbar abnormality detection systems.

Artificial-intelligence-based approaches have achieved great success in different domains, like image processing, image segmentation, text analysis, etc. [15,16]. In particular, medical image analysis, disease prognosis and detection, and medical data analysis have witnessed a great deal of success using such methods [17,18,19]. Machine learning and deep learning models have been widely adopted for disease detection and prediction. Several CAD approaches have been presented in the past few years for LSS diagnosis [20,21,22]. These studies show that convolutional neural networks (CNNs), which are particularly good at processing image data, show promising results for LSS detection. CNN models are widely applied in combination with medical imaging modalities such as MRI and CT, with a high success rate. However, current LSS detection models suffer from low accuracy and require further investigation and improvement. In this regard, this study makes the following contributions.

- This study aims to automatically diagnose the types of foraminal LSS by analyzing axial MRI images. In this regard, a custom CNN architecture is proposed.
- For automated spine labeling and foraminal stenosis grading, this method is trained using large-scale data.
- Multi-input data are generated for single-region and multiregion LSS classification. Multiregion data include normal, mild, moderate, and severe classes.
- Assessment of the proposed model is carried out using quantitative and qualitative methods on a publicly available MRI dataset. Afterward, the results are compared to those of existing approaches.

In this study, Section 2 presents the proposed approach, a description of the dataset used in this study, and the details of the proposed CNN model. Experimental results and discussions are presented in Section 3. Conclusions are presented in Section 4.

## 2. Materials and Methods

This section describes the proposed approach for lumbar spine stenosis detection, the proposed CNN model used for experiments, the dataset used in the study, and performance evaluation metrics.

### 2.1. Overview of Proposed System

**Figure 1:**
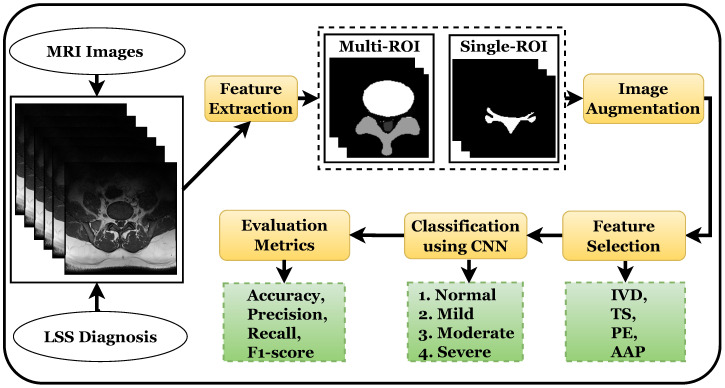
Flow chart of a standard CAD system for diagnosis of lumbar spine stenosis.

The proposed system uses a deep learning approach for feature extraction that classifies the foraminal or lateral stenosis into normal, mild, moderate, and severe spine stenosis using a CNN, as presented in Figure 1. Several image preprocessing techniques are also used to improve the model’s performance.

#### 2.1.1. Description of Lumbar Spine MR Image Dataset

The dataset [23] used in this study is associated with a clinical study of 515 patients suffering from back pain symptoms. The data were collected and analyzed by experienced radiologists and contain notes regarding the analyzed characteristics and conditions of the lower back, as well as the presence of disease. The data of each patient data are associated with one or more MRI studies. Each study contains slices (i.e., individual images taken from a either sagittal or axial view) of the lowest three vertebrae and the three lowest IVDs. The axial view slices are mainly taken from these last three IVDs. In most cases, the total number of slices in the axial view ranges from 12 to 15 [10]. The best images were extracted from the three lowest vertebrae in the axial view of IVDs, like L3–D3, L4–D4, and L5–D5. The image resolution is 320 × 320 pixels. Image pixels have a precision of 12 bits per pixel, but grayscale pixels have a precision of 8 bits per pixel, which is greater. Further grading was performed by an expert radiologist based on the clinical notes provided within the dataset; MRI lumbar spine stenosis scans were evaluated as normal, mild, moderate, or severe. This evaluation was based on the observer’s characteristics, as well as the condition of the lumbar spine and the presence of disease, such as bone marrow disease, endplate degeneration, IVD bulges, TS compression, central vs. FS, annular tears, scoliosis, endplate defects, facet joint, and ligamentum flavum hypertrophy and spondylosis.

#### 2.1.2. Region of Interest Extraction

To extract the desired region, a unique identification (ID) was allocated to each region, such as 1 (IVD), 2 (PE), 3 (TS), and 4 (AAP), as described in [9]. These regions were extracted by their associated IDs. Two datasets were created from a single dataset, each with a distinct region based on its ID. In the first dataset, IVD, PE, AAP, and TS were chosen, whereas, in the second dataset, only single-ROI (AAP) was extracted. LSS causes AAP compression, which puts pressure on the central spinal canal or nerve roots because stenosis can occur anywhere along the AAP. A general practitioner primarily evaluates three distances in the AAP to diagnose LSS: the AAP diameter and the left and right foramen widths.

#### 2.1.3. MR Image Cropping

Cropping a significant ROI of each image is a method used as a processing step for image data with both height and width dimensions. Additionally, random cropping is used to minimize the size of the input. The computation time is heavily influenced by the image resolution. The resolution of the available dataset is 320 × 320 pixels. The images were cropped with a resolution of 240 × 240 pixels. Despite the lack of considerable difference in image size, the computing time was significantly impacted by the reduced size. The primary images were cropped to a uniform image while maintaining their aspect ratio.

#### 2.1.4. Dataset Augmentation Techniques

Deep learning models require large datasets to reduce the possibilities of overfitting and provide better results [24,25]. Medical image analysis domains, on the other hand, do not have access to such big datasets. Consequently, depending on the need to expand the amount of data, different augmentation techniques have been used in the existing literature [26,27,28]. In this study, the size of the training dataset was increased using these techniques.

The left, right, top, or bottom translation of images is chosen to prevent positional bias in the data while maintaining the image dimensions. Translation was used on the training dataset, whereas the testing dataset was solely made up of the original images. Zoom augmentation casually enlarges the image and adds new pixel values to the surrounding area. The zoom range is [1 − value, 1 + value], which means that if the range is 0.9 to 0.7, the image is 90 to 70% zoomed-in, and if the range is 1.1 to 1.3, the image is 110 to 130% zoomed-out. For random rotation augmentation, the images are rotated right or left on an axis of 1${}^{\circ }$ to 359${}^{\circ }$. The rotation degree parameter, which is set between 1 and 20 or −1 to −20, has a remarkable effect on the safety of rotation augmentations.

#### 2.1.5. Architecture of the Proposed CNN Model

**Figure 2:**
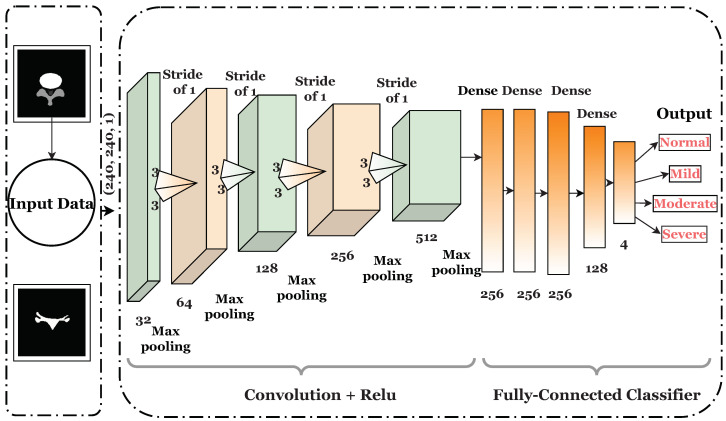
CNN architecture for image classification.

CNN is the most well-known and widely used type of algorithm in the field of deep learning, particularly for image processing [29,30,31,32]. A CNN automatically finds significant features without the need for human intervention. A CNN is a multilayered model with several convolution layers leading to subsampling (pooling) layers and fully connected layers at the end. Figure 2 shows the architecture of the proposed CNN for image categorization.

The first layer of the proposed architecture is a convolution layer that detects and extracts patterns and features from an input image. It keeps the pixels together by learning image patterns via small squares of input data. In a CNN model, the input (*x*) of each layer is structured in three aspects: height, width, and depth (or m×m×r), where the height (*m*) equals the width of 240 × 240 pixels. The channel number is another term for depth. The depth (*r*) of a grayscale image is set as 1. Each convolutional layer comprises a number of feature maps (filters) that are designated by *k*, with three dimensions (n×n×q), related to the input image.

Filters learn patterns such as edge detection, texture, corners, blur, and sharpening. In the next step, the stride shifts the number of pixels per step on the input matrix. Here, the stride (1,1) passes the filters as 1 pixel gradually. The model padding parameter is set as ‘same’. If the filter occasionally does not fit well with the input image, then there is the choice of padding so that it fits according to the requirement; otherwise valid padding drops, are used for the part where it does not fit perfectly.

The convolution and pooling layer is accountable for reducing the spatial size of the features convolved by applying a filter that also decreases the computational requirement to process the data by reducing dimensionality. Furthermore, it is useful for extracting features, since augmentation techniques are applied to data that have been rotated and shifted around without losing information. Furthermore, it maintains the process of efficient model training. The rectified linear unit (ReLU) activation function is utilized to map the input to the output. ReLU is the most-often-used function in the CNN context, since it reduces the model’s computational complexity.

In a fully connected layer that is located at the end of the CNN architecture, the matrix is turned into a vector, similar to a neural network. We linked these features together to form a model, since each neuron in the input layer is connected to the neurons in the output layer. Ultimately, we used a softmax activation function to categorize the outputs as normal, mild, moderate, or severe. Complete architectural details of the proposed CNN model are presented in Table 1.

### 2.2. Performance Evaluation

A range of evaluation criteria was employed to examine how well the model performed on augmented and non-augmented datasets with varied data values. These measures include class-specific metrics, as well as performance metrics, like accuracy, precision, sensitivity, and F1 score, which provide us with specific values to compare the algorithm efficiently. The following equations were used for these metrics:

(1) $$Accuracy=\frac{TP+TN}{TP+TN+FP+FN}$$

(2) $$Precision=\frac{TP}{TP+FP}$$

(3) $$Recall=\frac{TP}{TP+FN}$$

(4) $$F1\;score=2*\frac{Precision*Recall}{Precision+Recall}$$

where *TP*, *TN*, *FP*, and *FN* refer to true positive, true negative, false positive, and false negative, respectively.

## 3. Results, Analysis, and Discussion

This section provides details of the experimental setup, results of the proposed approach, and discussions of the results.

### 3.1. Experimental Setup

The proposed model was evaluated on a system with a Windows 10 operating system, 8 GB of RAM, and a Core i5 (3.6 GHz) CPU. Google Colab Pro was acquired with 38 GB of RAM and TPU processing. Python programming was utilized for experiments. The training took an average of 810 s (3 s/steps) for each image.

### 3.2. Data Preparation

**Figure 3:**
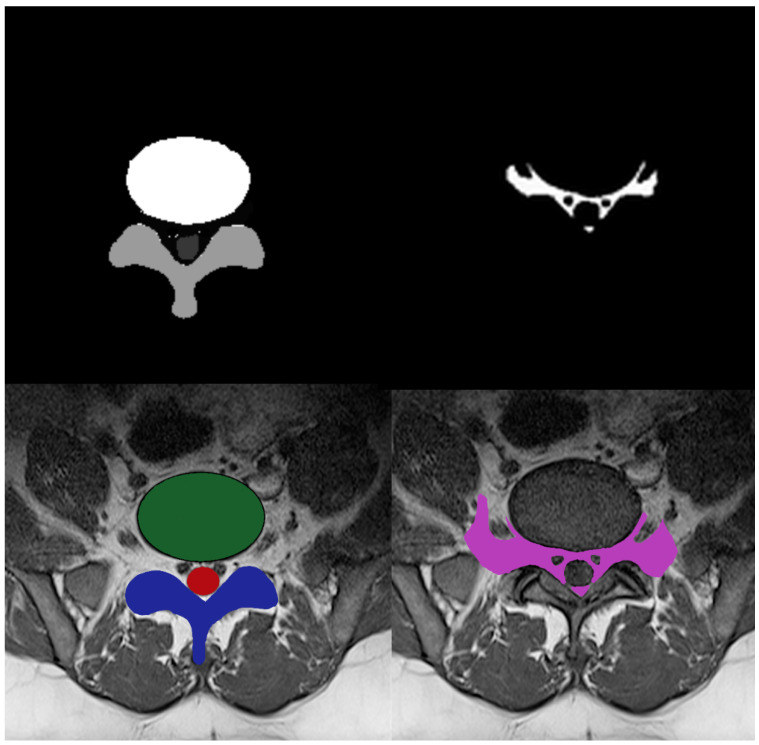
Four-class manual segmentation mask.

The initial step in using deep learning models is to prepare the training data for the classifiers. The deep learning approach is tremendously data-hungry because it also incorporates representation learning [33]. Multi-ROI and single-ROI training datasets were employed in our experiment. The first mask shown in Figure 3, contained IVD, PE, and TS, but the second mask only included the AAP region, so stenosis diagnosis could be performed after measuring this region.

To improve model performance, training was performed using various augmented sets of data including 5 k, 10 k, and 12.5 k, and testing was performed on 330 labeled images. The training was carried out using an 80:20 ratio; the classifier was trained on 80% and tested on 20% of the data. The training and testing ratio was chosen randomly, which was found to be most effective in prior research [10].

The model takes modest steps to reduce the negative gradient of the loss function, which is specified as the categorical cross-entropy probability distribution of each class. The learning rate parameter, which is 0.001, alters the step size, and the Adam optimizer is utilized. In the 12.5 k dataset, the batch size utilized for training was 256 per image, for a total of 34 epochs. A batch size of 128 with 34 epochs was applied in the 10 k dataset, with a batch size of 64 with 200 epochs in the case of the 5 k dataset.

The proposed model outperformed the compared models because its pruning neural networks reduced computational complexity and training inputs to some extent. The model was trained using 34 epochs instead of 100 epochs with a GPU, as the model took less than 12 h to complete the training process and produce the results, whereas prior models [34] took two or more days to complete the training process. After these epochs, the model showed no improvement in performance.

### 3.3. Results Using 12.5 K Dataset

**Figure 4:**
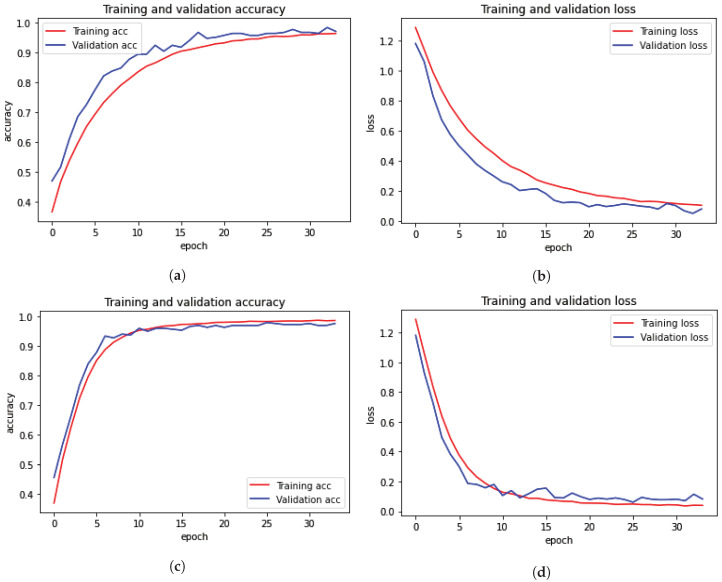
Training and validation loss and accuracy with 12.5 k data samples. (**a**) Training and validation accuracy for multi-ROI dataset. (**b**) Training and validation loss for multi-ROI dataset. (**c**) Training and validation accuracy for single-ROI dataset. (**d**) Training and validation loss for single-ROI dataset.

Plots of the model’s training and testing accuracy plot are shown in Figure 4; the model was trained for 34 epochs for both multi-ROI and single-ROI datasets. The model learns rapidly as training data are fed into it, and the training curve steadily increases until all epochs are completed. As epochs increase, the validation accuracy is increased in lockstep with the training accuracy curve. To compute model loss from the plot, the model travels through the same epochs. The model’s validation loss is significant at the start of the epochs, but as the number of epochs increases, the loss decreases.

Table 2 demonstrates multi-ROI and single-ROI classification reports of classes using the CNN, with precision of 0.94, recall of 0.92, and F1 score of 0.93 for the mild class. Precision and recall for the moderate class are 0.96 and 0.98, respectively. Precision for the normal class is recorded as 1.00. The average multiclass accuracy of the model for the multi-ROI dataset is 0.97. The macro average and weighed average were also calculated for precision, recall, and F1 score as 0.97 each for multi-ROI data.

The other half of Table 2 shows precision and recall values of 0.96 for the moderate class and precision of 0.97 for the normal class. The F1 score and recall for the normal class and F1 score for severe class are both 0.98. For the mild class, the precision, recall, and F1 score are each 0.99. The overall accuracy score of the model for all single-ROI is 0.97.

### 3.4. Results Using 10 K Dataset

**Figure 5:**
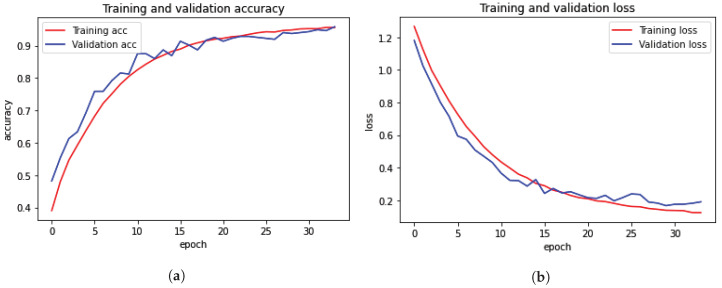

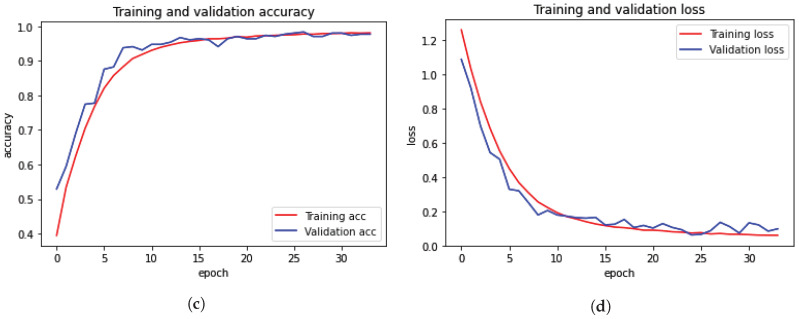
Training and validation loss and accuracy for grading of LSS with 10 k data samples. (**a**) Training and validation accuracy for multi-ROI dataset. (**b**) Training and validation loss for multi-ROI dataset. (**c**) Training and validation accuracy for single-ROI dataset. (**d**) Training and validation loss for single-ROI dataset.

The CNN accuracy plot in Figure 5 shows the training process of the model for 34 epochs for both the multi-ROI and single-ROI datasets. The model learns gradually as training progresses, although its accuracy graphs in both datasets exhibit variance towards the conclusion. Multi-ROI data training and validation accuracy fluctuate, whereas the accuracy of single-ROI training and validation increases steadily as the number of epochs increases. The model traverses the same epochs to compute model loss from the plot. The validation loss of the model was initially substantial, but as the number of epochs increased, the quantity of loss dropped. The results of the proposed model are varied in comparison to previously reported results.

Table 3 displays multi-ROI and single-ROI classification reports of classes using the CNN, which shows a precision of 0.92, recall of 0.96, and F1 score of 0.94, for the moderate class using multi-ROI data. The precision for the severe class is 0.94, while the F1 score for the mild and moderate classes are also 0.94. The precision, recall, and F1 score for the normal class are 0.98, 1.00 and 0.99, respectively. The average accuracy for the multi-ROI data using 10 k data is 0.96, which is slightly lower than that achieved using 12.5 k data.

The weighted average for precision, recall, and F1 scores is 0.98 each for single-ROI data using 10 k data. The precision for the normal class is 0.97, while the recall and F1 scores for the severe class and F1 score for normal class is 0.98. For the moderate class, precision, recall, and F1 scores are 0.96 each. The CNN model achieves a 0.98 accuracy score for a single-ROI dataset with 10 k data.

### 3.5. Results Using 5 K Dataset

**Figure 6:**
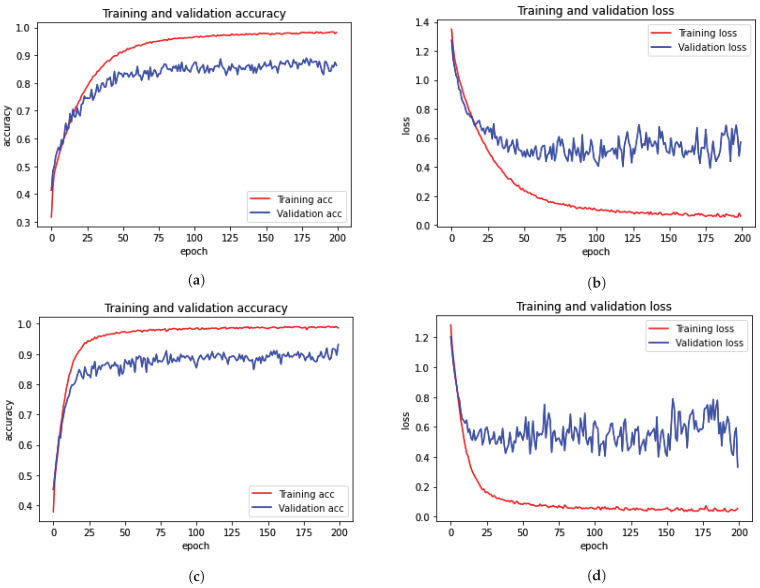
Training and validation loss for grading of LSS with 5 k data samples. (**a**) Training and validation accuracy for multi-ROI dataset. (**b**) Training and validation loss for multi-ROI dataset. (**c**) Training and validation accuracy for single-ROI dataset. (**d**) Training and validation loss for single-ROI dataset.

Figure 6 shows the training and validation accuracy and loss for the models using 200 epochs for both multi-ROI and single-ROI datasets. In this scenario, the model yields the lowest results compared to the other datasets. The model learns slowly as training proceeds, with the number of epochs increasing. With the increase in epochs, the training accuracy outperforms validation accuracy. Both datasets have a validation accuracy of 86.30% for multi-ROI and 93.15% for single-ROI. To depict the model loss, it went through the same number of epochs. The validation loss of the model was initially significant, but as the number of epochs increased, the amount of loss decreased slightly.

Table 4 illustrates the multi-ROI and single-ROI classification report produced by the CNN model, yielding a precision of 0.74 and an F1 score of 0.79 for the moderate class. The precision, recall, and F1 scores for the mild class are all 0.81, whereas those for normal and moderate classes are 0.84 and 0.85, respectively. The precision and recall for severe and normal classes are 0.86, 0.89, and 0.96 and 0.84, respectively.

Results for the single-ROI dataset indicate that for the normal and moderate classes, the recall and precision score is 0.86. The precision for the severe class is 0.89, while the recall and F1 scores are 0.99 and 0.94, respectively. In terms of accuracy, the CNN model achieves 0.86 and 0.92 accuracy scores for all multi-ROI and single-ROI classes, respectively. This performance is substantially lower compared to results achieved using a 12.5 k dataset.

### 3.6. Performance Comparison Using Different Dataset Sizes

Precision, recall, F1 score, and accuracy were calculated to compare the results of different dataset values, as presented in Table 5. For LSS detection, superior performance is obtained when a 12.5 k dataset is used with the proposed CNN model, achieving an accuracy score of 0.97, which is superior to that achieved when using both 10 k and 5 k datasets.

**Figure 7:**
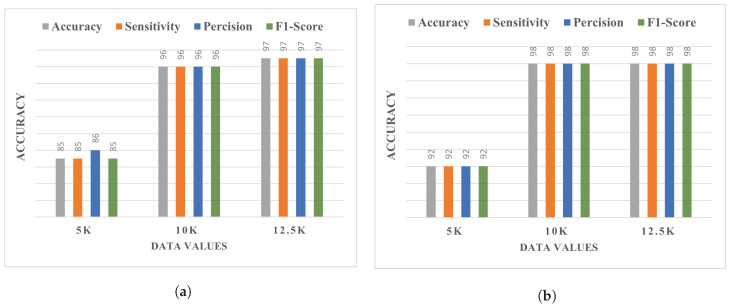
Comparative analysis of classification with different dataset values using the (**a**) Multi-ROI and (**b**) Single-ROI datasets.

Figure 7 provides a visual illustration of the performance comparison of the proposed CNN model using different dataset sizes. It can be observed that using the 5 k dataset, the model shows significantly low performance compared to the 10 k and 12.5 k datasets. The achieved performance slightly differs between the 10 k and 12.5 k datasets, and the best performance is obtained using the 12.5 k dataset.

### 3.7. Performance Comparison for Augmented vs. Non-Augmented Data

Deep learning models are data-intensive and require significantly larger datasets to obtain complex features to achieve improved performance. The original dataset size was smaller, and the CNN model could not be trained well to achieve satisfactory accuracy for LSS detection. Consequently, we performed data augmentation to resolve this issue.

**Figure 8:**
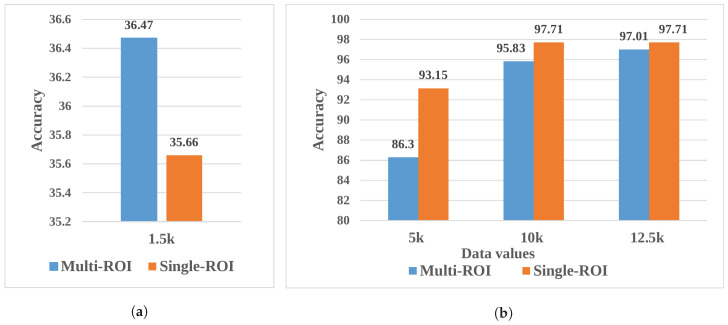
Comparative analysis of accuracy. (**a**) Non-augmented multi-ROI vs. single-ROI dataset. (**b**) Augmented multi-ROI vs. single-ROI dataset.

Table 6 and Figure 8 show comparisons of the results obtained using the original dataset with those achieved using the augmented dataset. When original data was used, the evaluation accuracy was relatively low, whereas, when augmented data were utilized, the results were much better. Comparably, the single-ROI results are better than those obtained with multi-ROI dataset.

### 3.8. Performance with Existing Approaches

In order to further advocating for the performance of the proposed model, a performance comparison with existing state-of-the-art approaches was also carried out. For this purpose, several approaches reported in existing literature were selected. For example, the methods proposed in [22,35] both use a CNN model for LSS detection, obtaining accuracies of 87.75% and 84.5%, respectively. Pretrained models have also been deployed, such as ResNet in [36] and VGG16 in [37]. Higher performance was reported in [38], with 94% accuracy, while the authors of [39] recently reported 95% accuracy. The results comparison presented in Table 7 indicates that the proposed model outperforms existing approaches.

### 3.9. Discussion

Over the last decade, several CAD approaches [41,42,43,44,45] have been investigated for their potential to address the challenges of spinal MRI interpretation and full automation the LSS diagnostic procedure, which could help to improve detection accuracy. In this regard, the CNN model is often employed with medical imaging modalities such as MRI and computed tomography (CT), with a high success rate. Several previously proposed approaches for neural foraminal stenosis disease detection using binary and multigraded (normal, mild, moderate, and severe) classification are discussed herein.

Among the most current diagnostic frameworks for LSS is that proposed by Natalia et al. [20], who used the SegNet model to automatically assess the area between the anterior and posterior (AAP) diameter and foraminal widths in MRI-, T1-, and T2-weighted composite images. Six ROIs were extracted after semantic segmentation, including intervertebral disc (IVD), posterior element (PE), and thecal sac (TS), as well as auxiliary ROIs, such as AAP and others. The contour evaluation technique was used to increase the accuracy of the segmentation result in specified ROIs. The results demonstrate a 96.7% diameter agreement with the expert. Similarly, Sartoretti’s classification [21] is based on a six-point grading system for detecting lumbar foraminal stenosis (FS) on MRI images of high resolution. Grade A has no FS. The superior, posterior, inferior, and anterior boundaries of the lumbar foramen are graded B, C, D, and E, respectively, indicating nerve root contact with surrounding anatomical structures. The existence of FS in the nerve root with morphological changes was graded F in this research, in which we employed sagittal high-resolution T1-weighted and T2-weighted MRI data from 101 subjects.

A study in regard to grading of CAD systems by Salehi et al. [22] showed that a CNN can be utilized to diagnose disc herniation using MRI images. A performance evaluation was carried out for normal, bulge, protrusion, and extrusion images. The experiment was performed on 2329 axial-view lumbar MRI datasets collected from a local medical center. Experimental results reported an 87.75% accuracy with data augmentation. Lu et al. [38] used the U-Net architecture of the CNN model to grade central and FS as normal, mild, moderate, or severe based on both sagittal and axial MRI images. A large-scale dataset of 22,796 was used, which included data from 4075 patients. An accuracy of 94% was reported for this study.

A different technique proposed by Han et al. [40] localizes six vertebrae and disc T12 to S1 using a deep multiscale multitask learning network (DMML-Net) that integrated into a full convolution network that grades the lumbar neural FS into normal and abnormal cases. The experimental setup included a dataset comprising 200 T1- and T2-weighted MRI images from 200 patients, achieving an accuracy of 84.5% using the proposed approach. An approach recently proposed by Hallinan et al. [35] is to classify neural foraminal stenosis into normal, mild, moderate, or severe classes using a deep learning CNN model that achieved 84.5% accuracy using a dataset of T2-weighted axial MRI images and T1-weighted sagittal MRI images from 446 patients.

Using a deep learning ResNet-50 model, multitask classification was performed in [36], which demonstrated the automated grading of lumbar disc herniation (LDH), lumbar central canal stenosis (LCCS), and lumbar nerve roots compression (LNRC) in lumbar axial MRIs. An internal test dataset and an external test dataset were used for classification systems with four graded levels (grade 0, grade 1, grade 2, and grade 3). A total of 1115 patients (1015 patients from the internal dataset and 100 patients from the external test dataset) were evaluated, and the best MRI slices were obtained. The efficiency of the model on the given datasets was evaluated using precision, accuracy, sensitivity, specificity, F1 scores, confusion matrices, receiver operating characteristics, and inter-rater agreement (Gwet k). On the internal test dataset, the overall grading accuracy for LDH, LCCS, and LNRC were 84.17%, 86.99%, and 81.21%, respectively. For the external test data, 74.16%, 79.65%, and 81.21% accuracy are reported for LDH, LCCS, and LNRC, respectively.

Bharadwaj et al. [46] utilized a V-Net model to segment the dural sac and IVD and localize the facet and foramen. Big transfer (BiT) models were trained for classification tasks. Multievaluation metrics including Cohen’s Kappa score were used for the dural sac and IVD. The authors used axial T2-weighted MRI images of the lumbar spine obtained between 2008 and 2019. The area under the receiver operator characteristic curve (AUROC) values used for the binary classification of facet and neural foraminal stenosis were 0.92 and 0.93, respectively. Sinan et al. [37] proposed an LSS-VGG16 and U-Net model that detects LSS in MR and CT images and achieved 87.70% classification accuracy on VGG16. A total of 1560 MR images were used with U-Net, with a 0.93 DICE score.

The authors of [47] a 3D LSS segmentation framework that enables the complete determination of the regions of the body that cannot be fully opened during LSS surgeries, particularly in the nerve roots. The spinal disc, canal, thecal sac, posterior element, and other regions and backgrounds in the image that are crucial for LSS were all segmented and divided into a total of six classes in MRI images. The intersection over union (IoU) metric was deployed for each class to assess the success of segmentation, since the canal had an IoU value of 0.61. The study employed T2 sequence lumbar MRI images of 300 LSS patients in the digital imaging and communications in medicine (DICOM) format.

Abhinav et al. [48] also recently presented a U-Net-dependent CNN model to segment the IVD, PE, TS, and AAP regions of LSS on an axial MRI dataset [10] and performed binary classification. The performance of the model was evaluated by IoU metrics. Since IVD is the simplest region to label and PE has a particular shape that resembles the letter Y, the values of regions like IVD, PE, and IoU vary between 0.80 and 1.0. And because AAP was the most challenging to identify, its IoU metric value is 0.6568, which is lower than that of the other regions.

Another innovative study [39] compared conventional and ultrafast methods and analyzed sagittal T1-weighted, T2-weighted, short-TI inversion recovery, and axial T2-weighted MRI images of 58 patients. Cohen’s kappa metrics were used to assess foraminal stenosis in axial images, and the results were provided in a multigraded classification. The accuracy obtained using this method was 95%.

In this study, we investigated LSS detection using a customized CNN model. We evaluated the algorithm’s performance using a variety of metrics. Experiments were conducted using two datasets and with and without augmentation techniques using different data values. Multi-ROI and single-ROI datasets with 5 k achieved the lowest results in terms of accuracy scores: 0.85 and 0.92, respectively. The cure of model accuracy shows that the model could be trained more to prevent underfitting and inflection because the model was not overlearned for the training set. Due to inadequate training, the model loss exhibits a divergence from the training curve, which indicates why the overall loss is large in the results of both datasets.

The two 10 k datasets achieved accuracy scores of 0.96 and 0.98, respectively. Figure 6 demonstrates that the trained model fit well; however, the validation curve is slightly unsatisfactory, owing to underfitting for the multi-ROI dataset, requiring more training data samples to improve accuracy. The model loss curve shows that training significantly decreased the loss, although it remains high during the initial epochs.

Table 5 illustrates that the accuracy is similar between the two 12.5 k datasets: 0.97 and 0.98, respectively. The model accuracy curve show in Figure 4 indicates that while model training performs well on a single-ROI dataset, results on a multi-ROI dataset might be further enhanced by adding more training data and by further reducing model loss.

Table 8 shows an analytical summary of the discussed research works. It can be observed that for LSS detection and segmentation, the models suffer from low accuracy. The CNN model and its variants were tested, yet no CNN technique was able to more accurately categorize LSS disease, necessitating the development of automatic methods that better classify the disease.

## 4. Conclusions

In this study, we proposed a technique to assist doctors in detecting and grading neural foraminal LSS using MRI images. Four regions of IVD, PE, TS, and AAP were selected as areas of focus in this study. For LSS detection, we proposed a customized CNN model and performed experiments using a publicly available lumbar spine dataset. The dataset consists of a back pain characteristics report annotated by expert radiologists and 515 patient MRI scan images of L3–L5 in axial view. Observed characteristics in annotated studies were further classified as normal, mild, moderate, or severe to investigate the reliability of the proposed deep-learning-based stenosis grading system. We constructed two datasets—a multi-ROI and single-ROI dataset—then trained the model on a variety of dataset values. Experiments were conducted using 5 k, 10 k, and 12.5 k datasets, which were produced using data augmentation. Experimental results indicate that better performance can be obtained using an augmented dataset. The best performance was achieved with a 12.5 k dataset for both single-ROI and multi-ROI datasets, showing 97.71% and 97.01% accuracy, respectively. Performance comparison with existing state-of-the-art approaches validated the superior performance of the proposed approach.

## Figures and Tables

**Table 1:** Summary of the proposed CNN model.

Layer	Feature Map	Filter	Strides	Padding	Activation
Convolution	32	3 × 3	1 × 1	Same	ReLU
Max Pooling	32	2 × 2	-	Same	Relu
Convolution	64	3 × 3	1 × 1	Same	ReLU
Max Pooling	64	2 × 2	-	Same	Relu
Convolution	128	3 × 3	1 × 1	Same	ReLU
Max Pooling	128	2 × 2	-	Same	Relu
Convolution	256	3 × 3	1 × 1	Same	ReLU
Max Pooling	256	2 × 2	-	Same	Relu
Convolution	512	3 × 3	1 × 1	Same	ReLU
Max Pooling	512	2 × 2	-	Same	ReLU
Fully Connected	256	-	-	-	ReLU
Fully Connected	256	-	-	-	ReLU
Fully Connected	256	-	-	-	ReLU
Fully Connected	128	-	-	-	ReLU
Fully Connected	4	-	-	-	Softmax

**Table 2:** Comparative results of multi-ROI and single-ROI with 12.5 k data.

ROI Type	Accuracy	Class	Precision	Recall	F1 Score
Multi-ROI dataset	0.97	Normal	1.00	0.99	0.99
		Mild	0.94	0.92	0.93
		Moderate	0.96	0.98	0.97
		Severe	0.97	0.97	0.97
		Macro avg.	0.97	0.96	0.96
		Weighted avg.	0.97	0.97	0.97
Single-ROI dataset	0.98	Normal	0.97	0.99	0.98
		Mild	0.99	0.99	0.99
		Moderate	0.96	0.96	0.96
		Severe	0.99	0.98	0.98
		Macro avg.	0.98	0.98	0.98
		Weighted avg.	0.98	0.98	0.98

**Table 3:** Comparative results of multi-ROI and single-ROI with 10 k data.

ROI Type	Accuracy	Class	Precision	Recall	F1 Score
Multi-ROI dataset	0.96	Normal	0.98	1.00	0.99
		Mild	0.99	0.89	0.94
		Moderate	0.92	0.96	0.94
		Severe	0.94	0.99	0.97
		Macro avg.	0.96	0.96	0.96
		Weighted avg.	0.96	0.96	0.96
Single-ROI dataset	0.98	Normal	0.97	0.99	0.98
		Mild	0.99	0.99	0.99
		Moderate	0.96	0.96	0.96
		Severe	0.99	0.98	0.98
		Macro avg.	0.98	0.98	0.98
		Weighted avg.	0.98	0.98	0.98

**Table 4:** Comparative results of multi-ROI and single-ROI datasets with 5 k data.

ROI Type	Accuracy	Class	Precision	Recall	F1 Score
Multi-ROI dataset	0.85	Normal	0.96	0.84	0.90
		Mild	0.81	0.81	0.81
		Moderate	0.74	0.85	0.79
		Severe	0.86	0.89	0.87
		Macro avg.	0.84	0.85	0.84
		Weighted avg.	0.86	0.85	0.85
Single-ROI dataset	0.92	Normal	0.99	0.86	0.92
		Mild	0.91	0.90	0.91
		Moderate	0.86	0.94	0.90
		Severe	0.89	0.99	0.94
		Macro avg.	0.91	0.92	0.92
		Weighted avg.	0.92	0.92	0.92

**Table 5:** Comparative results of multi-ROI and single-ROI datasets with different dataset sizes.

ROI Type	Data Values	Precision	Recall	F1 Score	Accuracy
Multi-ROI dataset	5 k	0.86	0.85	0.85	0.85
	10 k	0.96	0.96	0.96	0.96
	12.5 k	0.97	0.97	0.97	0.97
Single-ROI dataset	5 k	0.92	0.92	0.92	0.92
	10 k	0.98	0.98	0.98	0.98
	12.5 k	0.98	0.98	0.98	0.98

**Table 6:** Comparison of overall dataset accuracy with different data values.

Data Type	Data Values	Multi-ROI Dataset	Single-ROI Dataset
Augmented data	5000	86.3	93.15
	10,000	95.83	97.71
	125,000	97.01	97.71
Non-Augmented Data	1545	36.47	35.66

**Table 7:** Performance comparison with state-of-the-art approaches.

Ref.	Model	Accuracy
[22]	CNN	87.75%
[38]	UNet	94%
[40]	DMML-Net	84.5%
[35]	CNN	84.5%
[36]	ResNet-20	LDH—84.17%,LCCS—86.99%,LNRC—81.21%
[37]	VGG16	87.70%
[39]	Different models	95%
Proposed	CNN	97.71% for singl-ROI with 12.5 k data97.01% for multi-ROI with 12.5 k data

**Table 8:** Analytical overview of the discussed research works.

Ref.	Model	Data	Results
[20]	SegNet	2D T1- and T2-weighted MRI images with 320 × 320 resolution	96.7% agreement with medical expert
[22]	CNN	High-resolution 3D MRI images	87.75% accuracy with data augmentation
[35]	CNN	2D T1- and T2-weighted MRI images	84.5% accuracy
[36]	ResNet-20	2D T2-weighted MRI images	LDH, LCCS, and LNRC accuracy of 84.17%, 86.99%, and 81.21%, respectively, for internal dataset.74.16%, 79.65%, and 81.21% accuracy for LDH, LCCS, and LNRC, respectively, using external dataset
[37]	VGG16	2D MRI images	87.70% accuracy
[38]	U-Net	High-resolution 2D MRI images with 512 × 512 resolution	94% accuracy
[46]	V-Net model	2D T2-weighted MRI images	Facet and neural foraminal stenosis AUC of 0.92 and 0.93, respectively
[39]	Different models	MRI T1-weighted, T2-weighted, short T1-inversion	95% accuracy
[40]	DMML-Net	High-resolution 2D T1- and T2-weighted MRI images with 512 × 512 resolution	84.5% accuracy

## Data Availability

Not applicable.

## References

[1] Raja A., Hoang S., Patel P., Mesfin F.B. (2017). Spinal Stenosis.

[2] Al Kafri A.S., Sudirman S., Hussain A.J., Fergus P., Al-Jumeily D., Al-Jumaily M., Al-Askar H. (2016). A framework on a computer assisted and systematic methodology for detection of chronic lower back pain using artificial intelligence and computer graphics technologies. Proceedings of the Intelligent Computing Theories and Application: 12th International Conference, ICIC 2016.

[3] Hoy D., March L., Woolf A., Blyth F., Brooks P., Smith E., Vos T., Barendregt J., Blore J., Murray C. (2014). The global burden of neck pain: Estimates from the Global Burden of Disease 2010 study. Ann. Rheum. Dis..

[4] Fatoye F., Gebrye T., Odeyemi I. (2019). Real-world incidence and prevalence of low back pain using routinely collected data. Rheumatol. Int..

[5] Wu A., March L., Zheng X., Huang J., Wang X., Zhao J., Blyth F.M., Smith E., Buchbinder R., Hoy D. (2020). Global low back pain prevalence and years lived with disability from 1990 to 2017: Estimates from the Global Burden of Disease Study 2017. Ann. Transl. Med..

[6] Hoy D., Bain C., Williams G., March L., Brooks P., Blyth F., Woolf A., Vos T., Buchbinder R. (2012). A systematic review of the global prevalence of low back pain. Arthritis Rheum..

[7] Versus Arthritis (2019). The State of Musculoskeletal Health. https://www.versusarthritis.org/about-arthritis/data-and-statistics/the-state-of-musculoskeletal-health/.

[8] Verbiest H. (1976). Neurogenic Intermittent Claudication: With Special Reference to Stenosis of the Lumbar Vertebral Canal.

[9] Natalia F., Meidia H., Afriliana N., Al-Kafri A., Sudirman S. Methodology to Determine Important-Points Location for Automated Lumbar Spine Stenosis Diagnosis Procedure. Proceedings of the 2019 International Conference on Intelligent Medicine and Health.

[10] Al-Kafri A.S., Sudirman S., Hussain A., Al-Jumeily D., Natalia F., Meidia H., Afriliana N., Al-Rashdan W., Bashtawi M., Al-Jumaily M. (2019). Boundary delineation of MRI images for lumbar spinal stenosis detection through semantic segmentation using deep neural networks. IEEE Access.

[11] Savigny P., Kuntze S., Watson P., Underwood M., Ritchie G., Cotterell M., Hill D., Browne N., Buchanan E., Coffey P. (2009). Low Back Pain: Early Management of Persistent Non-Specific Low Back Pain.

[12] Nguyen H.S., Doan N., Shabani S., Baisden J., Wolfla C., Paskoff G., Shender B., Stemper B. (2016). Upright magnetic resonance imaging of the lumbar spine: Back pain and radiculopathy. J. Craniovertebral Junction Spine.

[13] Bhargavan M., Sunshine J.H., Schepps B. (2002). Too few radiologists?. Am. J. Roentgenol..

[14] Royal College of Radiologists (2016). Clinical radiology UK Workforce Census 2015 Report. https://www.rcr.ac.uk/publication/clinical-radiology-uk-workforce-census-2015-report.

[15] Reshi A.A., Ashraf I., Rustam F., Shahzad H.F., Mehmood A., Choi G.S. (2021). Diagnosis of vertebral column pathologies using concatenated resampling with machine learning algorithms. PeerJ Comput. Sci..

[16] Jalal N., Mehmood A., Choi G.S., Ashraf I. (2022). A novel improved random forest for text classification using feature ranking and optimal number of trees. J. King Saud-Univ.-Comput. Inf. Sci..

[17] Rupapara V., Rustam F., Aljedaani W., Shahzad H.F., Lee E., Ashraf I. (2022). Blood cancer prediction using leukemia microarray gene data and hybrid logistic vector trees model. Sci. Rep..

[18] Umer M., Ashraf I., Ullah S., Mehmood A., Choi G.S. (2022). COVINet: A convolutional neural network approach for predicting COVID-19 from chest X-ray images. J. Ambient. Intell. Humaniz. Comput..

[19] Shafi I., Din S., Khan A., Díez I.D.L.T., Casanova R.d.J.P., Pifarre K.T., Ashraf I. (2022). An effective method for lung cancer diagnosis from ct scan using deep learning-based support vector network. Cancers.

[20] Natalia F., Meidia H., Afriliana N., Young J.C., Yunus R.E., Al-Jumaily M., Al-Kafri A., Sudirman S. (2020). Automated measurement of anteroposterior diameter and foraminal widths in MRI images for lumbar spinal stenosis diagnosis. PLoS ONE.

[21] Sartoretti E., Wyss M., Alfieri A., Binkert C.A., Erne C., Sartoretti-Schefer S., Sartoretti T. (2021). Introduction and reproducibility of an updated practical grading system for lumbar foraminal stenosis based on high-resolution MR imaging. Sci. Rep..

[22] Salehi E., Khanbare S., Yousefi H., Sharpasand H., Sojoodi Sheyjani O. Deep Convolutional Neural Networks for Automated Diagnosis of Disc Herniation on Axial MRI. Proceedings of the 2019 Scientific Meeting on Electrical-Electronics Biomedical Engineering and Computer Science (EBBT).

[23] Sudirman S., Kafri A.A., Natalia F., Meidia H., Afriliana N., Al-Rashdan W., Bashtawi M., Al-Jumaily M. (2019). Lumbar Spine MRI Dataset. Mendeley Data.

[24] Srivastava N., Hinton G., Krizhevsky A., Sutskever I., Salakhutdinov R. (2014). Dropout: A simple way to prevent neural networks from overfitting. J. Mach. Learn. Res..

[25] Xu Q., Zhang M., Gu Z., Pan G. (2019). Overfitting remedy by sparsifying regularization on fully-connected layers of CNNs. Neurocomputing.

[26] Shorten C., Khoshgoftaar T.M. (2019). A survey on image data augmentation for deep learning. J. Big Data.

[27] Shafique R., Rustam F., Choi G.S., Díez I.d.l.T., Mahmood A., Lipari V., Velasco C.L.R., Ashraf I. (2023). Breast cancer prediction using fine needle aspiration features and upsampling with supervised machine learning. Cancers.

[28] Hirahara D., Takaya E., Takahara T., Ueda T. (2020). Effects of data count and image scaling on deep learning training. PeerJ Comput. Sci..

[29] Krizhevsky A., Sutskever I., Hinton G.E., Pereira F., Burges C.J.C., Bottou L., Weinberger K.Q. (2012). ImageNet Classification with Deep Convolutional Neural Networks. Advances in Neural Information Processing Systems.

[30] Zhou D.X. (2020). Theory of deep convolutional neural networks: Downsampling. Neural Netw..

[31] Li G., Zhang M., Li J., Lv F., Tong G. (2021). Efficient densely connected convolutional neural networks. Pattern Recognit..

[32] Gu J., Wang Z., Kuen J., Ma L., Shahroudy A., Shuai B., Liu T., Wang X., Wang G., Cai J. (2018). Recent advances in convolutional neural networks. Pattern Recognit..

[33] Karimi H., Derr T., Tang J. (2019). Characterizing the decision boundary of deep neural networks. arXiv.

[34] Al-Kafri A. (2019). A Machine Learning and Computer Assisted Methodology for Diagnosing Chronic Lower Back Pain on Lumbar Spine Magnetic Resonance Images. Ph.D. Thesis.

[35] Hallinan J.T.P.D., Zhu L., Yang K., Makmur A., Algazwi D.A.R., Thian Y.L., Lau S., Choo Y.S., Eide S.E., Yap Q.V. (2021). Deep learning model for automated detection and classification of central canal, lateral recess, and neural foraminal stenosis at lumbar spine MRI. Radiology.

[36] Su Z.H., Liu J., Yang M.S., Chen Z.Y., You K., Shen J., Huang C.J., Zhao Q.H., Liu E.Q., Zhao L. (2022). Automatic grading of disc herniation, central canal stenosis and nerve roots compression in lumbar magnetic resonance image diagnosis. Front. Endocrinol..

[37] Altun S., Alkan A., Altun İ. (2023). LSS-VGG16: Diagnosis of Lumbar Spinal Stenosis With Deep Learning. Clin. Spine Surg..

[38] Lu J.T., Pedemonte S., Bizzo B., Doyle S., Andriole K.P., Michalski M.H., Gonzalez R.G., Pomerantz S.R., Doshi-Velez F., Fackler J., Jung K., Kale D., Ranganath R., Wallace B., Wiens J. (2018). Deep Spine: Automated Lumbar Vertebral Segmentation, Disc-Level Designation, and Spinal Stenosis Grading using Deep Learning. Proceedings of the 3rd Machine Learning for Healthcare Conference.

[39] Fujiwara M., Kashiwagi N., Matsuo C., Watanabe H., Kassai Y., Nakamoto A., Tomiyama N. (2023). Ultrafast lumbar spine MRI protocol using deep learning–based reconstruction: Diagnostic equivalence to a conventional protocol. Skelet. Radiol..

[40] Han Z., Wei B., Leung S., Nachum I.B., Laidley D., Li S. (2018). Automated pathogenesis-based diagnosis of lumbar neural foraminal stenosis via deep multiscale multitask learning. Neuroinformatics.

[41] Ruiz-España S., Arana E., Moratal D. (2015). Semiautomatic computer-aided classification of degenerative lumbar spine disease in magnetic resonance imaging. Comput. Biol. Med..

[42] Alomari R.S., Chaudhary V., Dhillon G. Computer Aided Diagnosis System for Lumbar Spine. Proceedings of the 4th International Symposium on Applied Sciences in Biomedical and Communication Technologies.

[43] Alomari R., Corso J.J., Chaudhary V., Dhillon G. (2011). Toward a clinical lumbar CAD: Herniation diagnosis. Int. J. Comput. Assist. Radiol. Surg..

[44] Nikravan M., Ebrahimzadeh E., Izadi M.R., Mikaeili M. (2016). Toward a computer aided diagnosis system for lumbar disc herniation disease based on MR images analysis. Biomed. Eng. Appl. Basis Commun..

[45] Ghosh S., Chaudhary V. (2014). Supervised methods for detection and segmentation of tissues in clinical lumbar MRI. Comput. Med. Imaging Graph..

[46] Bharadwaj U.U., Christine M., Li S., Chou D., Pedoia V., Link T.M., Chin C.T., Majumdar S. (2023). Deep learning for automated, interpretable classification of lumbar spinal stenosis and facet arthropathy from axial MRI. Eur. Radiol..

[47] Altun S., Alkan A. (2023). LSS-net: 3-dimensional segmentation of the spinal canal for the diagnosis of lumbar spinal stenosis. Int. J. Imaging Syst. Technol..

[48] Shukla A., Bhardwaj S., Singh M. (2023). Segmentation for Lumbar Spinal Stenosis Using Convolutional Neural Networks. Procedia Comput. Sci..

